# A CSI Approach Incorporating Recursive Eigenfunction Expansion for Efficient Microwave Imaging of Objects Embedded in Arbitrarily Shaped Multilayer Cylinders

**DOI:** 10.3390/s26134134

**Published:** 2026-07-01

**Authors:** Birol Aslanyürek, Tolga Ulaş Gürbüz

**Affiliations:** 1 Department of Mathematical Engineering, Yildiz Technical University, 34210 Istanbul, Türkiye; baslan@yildiz.edu.tr; 2 Department of Computer Engineering, Gaziantep University, 27310 Gaziantep, Türkiye

**Keywords:** contrast source inversion, multilayer cylinder, multilayer Green’s function, recursive algorithms, eigenfunction expansion method, arbitrarily shaped interfaces

## Abstract

Microwave imaging of objects embedded in multilayer cylindrical structures is of practical importance in applications where inaccessible targets are surrounded by a known inhomogeneous host. In such problems, incorporating the known multilayer structure into the background model can improve reconstruction accuracy and reduce the complexity of the inverse problem. This paper presents an efficient imaging method for dielectric objects embedded in two-dimensional multilayer cylindrical structures with arbitrarily shaped layer boundaries. The proposed approach integrates the contrast source inversion method with a recursive eigenfunction expansion technique for noncircular geometries. The known multilayer host is treated as the background medium, while the inversion is restricted to the embedded scatterers. The recursive formulation is derived to compute the inhomogeneous-background Green’s function and the required cell-integrated Green’s functions in a semi-analytical and discretization-free manner. Numerical results suggest that the method is capable of providing satisfactory reconstructions of embedded objects under various host configurations, including cases with a Perfect Electric Conductor (PEC) core. Comparisons with Method of Moments reference solutions confirm the accuracy of the forward modeling and the reliability of the inversion, while demonstrating a significant reduction in computational cost.

## 1. Introduction

Microwave imaging of objects embedded in inhomogeneous environments has been widely investigated for a variety of applications including biomedical diagnostics [1,2], subsurface sensing and ground-penetrating radar (GPR) [3], through-the-wall imaging [4], and the non-destructive evaluation of civil and industrial cylindrical components [5,6]. The common goal is to retrieve the location, shape or constitutive parameters of inaccessible targets using measured fields generated by the interaction of electromagnetic waves with the medium. In many of these scenarios, the targets are embedded in a known host such as a stratified ground, a multi-walled building, a cylindrical pipe, or a layered biological tissue. It is well known that exploiting this a priori knowledge may improve the accuracy, stability, and convergence of the reconstruction [7,8]. Such information can be used in different ways, for example as an initial estimate, as a regularization constraint, or by incorporating the host directly into an inhomogeneous background model. This has motivated the development of dedicated forward and inverse solvers tailored to specific geometries. A considerable body of these studies has focused on imaging problems involving multilayer configurations.

The majority of multilayer imaging studies consider planar multilayer or half-space backgrounds. Some examples of such studies include Born-type and distorted Born iterative methods [9], finite-element-based subsurface inversion in two-layered environments with planar and non-planar air-soil interfaces [10], nonlinear inversion enhanced with synthetic-aperture processing [11,12], and dedicated schemes for rough interfaces [13,14]. Comprehensive reviews are available in [3].

A second class of multilayer problems, more closely related to the present work, consists of cylindrically layered configurations in which the host medium is composed of layers with canonical cross-sectional boundaries such as circles or ellipses, and the embedded targets are reconstructed within the host medium. In this context, dielectric parameters and velocity profiles inside multilayer elliptic pipelines are retrieved in [5], Born approximation is applied for the reconstruction of arbitrary cross-section bodies in a lossy circular cylinder with a resistive boundary in [15], the point spread function of the linear inverse scattering problem for a dielectric cylinder background is evaluated in [16] to estimate the achievable resolution. Such formulations are effective because they exploit the separability of the geometry, but they are intrinsically limited to circularly or elliptically layered hosts.

When the layer interfaces are arbitrarily shaped, a straightforward approach within an inversion algorithm is to use the known multilayer profile only as an initial estimate and reconstruct the entire configuration, including both the host and the embedded targets, simultaneously. A more effective strategy, however, is to incorporate the known multilayer host directly into the background model. In this case, the inversion is performed only over the embedded objects. This strategy preserves the available a priori information throughout the iterative process, reduces the range of contrast values to be recovered by formulating the inversion in terms of the embedded objects, and avoids forcing the algorithm to reconstruct the high-contrast boundaries of the host. Within this framework, general inversion schemes developed for inhomogeneous backgrounds [17,18,19] can, in principle, be employed for arbitrarily shaped multilayer structures. In [17], embedded objects are reconstructed by using a subspace-based optimization method in an inhomogeneous background. In [18], the inverse problem associated with brain strokes is solved through a procedure using Tikhonov regularization. However, the approaches in [17,18] require the background Green’s function to be obtained numerically. In particular, ref. [17] employs the finite-element method (FEM) to simultaneously compute the Green’s function at all discrete points, whereas in [18] the Green’s function of the inhomogeneous medium is obtained through the Method of Moments (MoM). Although such approaches are accurate and general, the computation of the inhomogeneous-background Green’s function by discretization-based numerical solvers is computationally burdensome, especially when fine volumetric or surface discretization of the entire host is required. On the other hand, ref. [19] avoids the explicit computation of the Green’s function of the inhomogeneous background. Instead, it employs the Green’s function of a homogeneous medium and images the perturbations with respect to the known background by using a procedure based on conjugate-gradient fast Fourier transform and fast multipole methods. This approach has considerably low computational complexity. Nevertheless, it requires a rectangular domain of interest containing the entire configuration and relies on careful tuning of several regularization parameters.

In this paper, we propose an efficient imaging method for objects embedded in two-dimensional multilayer cylindrical structures with arbitrarily shaped layer boundaries. The proposed method avoids some of the main drawbacks of general inversion schemes for inhomogeneous backgrounds. More specifically, the proposed method does not rely on computationally burdensome discretization-based numerical solvers to obtain the background Green’s function. Moreover, unlike approaches that require a rectangular domain of interest enclosing the entire configuration, the reconstruction domain can be restricted to the region in which the embedded objects are sought. In this study, the problem is treated within the contrast source inversion (CSI) [20] framework. CSI is an effective iterative inversion method that accounts for the inherent nonlinearity of the inverse scattering problem without explicitly linearizing it. In addition to the standard CSI formulation, various extensions have been developed for different inverse scattering scenarios [21,22]. Here, we adapt the standard CSI to the present problem by restricting the inversion process to the contrast with respect to the known multilayer background. For this purpose, a recursive version of the previously proposed eigenfunction expansion method for noncircular surfaces (EE-NCS) [23,24] is developed and employed to evaluate the inhomogeneous-background Green’s function and the cell integrals of this Green’s function required in the CSI discretization. These quantities are computed once and reused throughout the CSI iterations.

The methodological contribution of the study can be summarized as follows. To the best of the authors’ knowledge, this is the first microwave imaging study developed specifically for objects embedded in multilayer cylindrical backgrounds with arbitrarily shaped layer boundaries. In contrast to a direct application of standard CSI, where the known multilayer profile is used as an initial estimate and the entire configuration is reconstructed, the proposed formulation incorporates the known multilayer host directly into the background model and restricts the inversion to the embedded objects. As demonstrated in the numerical examples, this strategy provides more accurate reconstructions than standard CSI applied with free space as the background and the known multilayer profile as the initial estimate. Furthermore, compared with other CSI-type inversion approaches that incorporate an inhomogeneous background through discretization-based numerical solvers such as MoM or FEM, the proposed method offers a computationally efficient alternative. This efficiency is achieved by embedding the recursive EE-NCS formulation, originally developed in our previous studies for forward scattering, into a complete CSI-based inverse imaging scheme. In particular, new recursive EE-NCS expressions are derived here to evaluate the inhomogeneous-background Green’s function and its cell-integrated counterparts required in the CSI discretization in a semi-analytical manner, without discretizing the entire multilayer host for the background Green’s function computation. We previously reported preliminary results for a simpler, non-recursive version of this approach applied to a single-layer cylindrical case in [25].

The accuracy of the proposed forward modeling and the reliability of the resulting inversion are validated against MoM reference solutions, and a series of numerical experiments demonstrates the capability of the present method to reconstruct the position, shape and electrical parameters of embedded objects under a variety of host configurations. It should be noted that the known general limitations of CSI and eigenfunction expansion also apply to the proposed method. Accordingly, the reconstruction performance may degrade for embedded scatterers with high contrast due to the increased nonlinearity of the inverse problem, whereas the accuracy of the EE-NCS-based forward modeling may be affected as the electrical size of the multilayer structure increases.

Throughout the paper, vectors and matrices are denoted by bold symbols, whereas scalar fields and functions are written in non-bold symbols. The time dependence is assumed to be of the form exp(-*iωt*) and is suppressed.

## 2. Statement of the Imaging Problem

**Figure 1:**
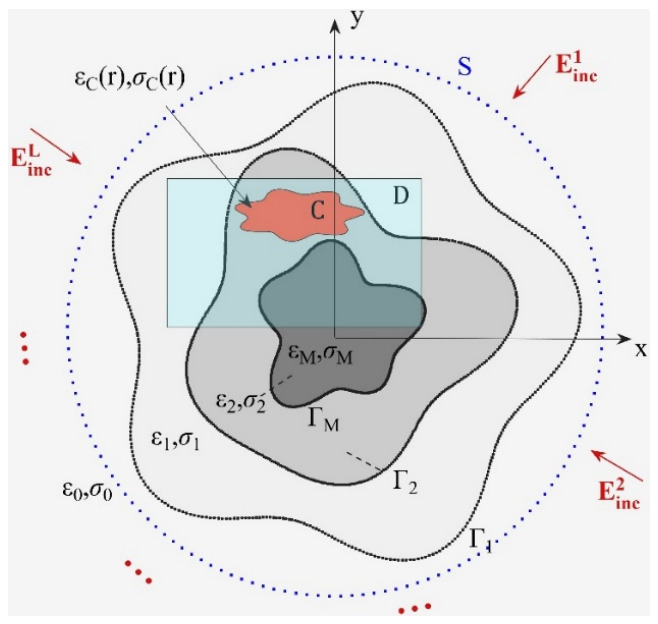
Configuration of the 2D inverse scattering problem. A dielectric target is embedded in an investigation domain *D* within a known multilayer cylindrical structure.

We consider a two-dimensional electromagnetic configuration that is invariant along the z-axis (see Figure 1). A dielectric target is embedded in a known multilayer cylindrical structure composed of M homogeneous, isotropic, and nonmagnetic layers separated by arbitrarily shaped boundaries. The background medium for the scattering problem is defined as the union of the M-layered cylindrical structure and the exterior free-space region. The dielectric permittivity and the conductivity of the mth layer (numbered from outside to inside) are $\varepsilon_{m}$ and $\sigma_{m}$, m=0,1,…,M, respectively. All magnetic permeabilities are equal to $\mu_{0}$. The interface between layer m−1 and layer m is denoted by $\Gamma_{m}$, m=1,2,…,M, and described in polar coordinates by $\rho =\;f_{m}\left(\phi \right)$, $\phi \in \left[0,2\pi \right)$, where each function $f_{m}$ is real-valued and piecewise smooth. The cross section C of the embedded object is assumed to be entirely contained in a prescribed investigation domain $D\subset R^{2}$. The object may be inhomogeneous, with constitutive parameters $\varepsilon_{C}\left(r\right)$, $\sigma_{C}\left(r\right)$ and $\mu_{0}$, where $r=\left(\rho ,\phi \right)$ is the position vector in polar coordinates. The multilayer structure with embedded object is illuminated successively by L single frequency time-harmonic transverse magnetic to the z-direction (TM_z_) polarized waves, whose electric fields are $E_{inc}^{l}\left(r\right)=u_{inc}^{l}\left(r\right)e_{z}$, l=1, 2,…, L, and for each illumination, the electric field vector is measured on a measurement curve S.

The objective is to reconstruct the spatial distributions of $\varepsilon_{C}\left(r\right)$ and $\sigma_{C}\left(r\right)$ using measured electric field data. To this end, the contrast function associated with the embedded object is introduced as

(1) $$\chi \left(r\right)=\frac{k^{2}(r)}{{k_{b}}^{2}\left(r\right)}-1,\;\;\;\;\;\;\;\;\;r\in D$$

where $k\left(r\right)$ and $k_{b}\left(r\right)$ denote the wavenumbers in the presence and absence of the object, respectively. Their squares are given by

(2) $${{k_{b}^{2}\left(r\right)=k_{m}^{2}=\omega }^{2}\varepsilon }_{m}\mu_{0}+i\omega \sigma_{m}\mu_{0},\;\;\;\;r\in layer\;m$$

and

(3) k2r=ω2εC(r)μ0+iωσC(r)μ0,     r∈ C                     kb2r,                                     r∉C.

Note that the contrast function $\chi \left(r\right)$ vanishes outside the object, since in that region the local material parameters coincide with those of the background layer. Inside C, $\chi \left(r\right)$ is determined by the object parameters $\varepsilon_{C}(r)$ and $\sigma_{C}(r)$.

Under TM_z_ polarization, the problem reduces to a scalar one in terms of the field function $u^{l}(r)$, which represents the total electric field vector $E^{l}(r)=u^{l}(r)e_{z}$ for the lth illumination. The total field can be decomposed as $u^{l}(r)=u_{b}^{l}(r)+u_{s}^{l}\left(r\right)$. Here, $u_{b}^{l}$ denotes the background field, i.e., the total field in the absence of the embedded object, whereas $u_{s}^{l}$ represents the field scattered by the embedded object. These fields satisfy the following system of integral equations

(4) $$u^{l}\left(r\right)=u_{b}^{l}\left(r\right)+\iint_{D}G_{b}\left(r;r^{\prime }\right){k_{b}}^{2}\left(r^{\prime }\right)\chi \left(r^{\prime }\right)u^{l}\left(r^{\prime }\right)dr^{\prime },\;\;r\in D$$

(5) $$u_{s}^{l}\left(r\right)=\iint_{D}G_{b}\left(r;r^{\prime }\right){k_{b}}^{2}\left(r^{\prime }\right)\chi \left(r^{\prime }\right)u^{l}\left(r^{\prime }\right)dr^{\prime },\;\;\;\;\;\;\;\;\;r\in S,$$

which are known as object and data equations, respectively. Here $G_{b}\left(r;r^{\prime }\right)$ is the Green’s function of the background which consists of the M-layered cylindrical structure and the exterior free-space region. The inverse problem is to retrieve the contrast distribution $\chi \left(r\right)$, and consequently the profiles $\varepsilon_{C}\left(r\right)$ and $\sigma_{C}\left(r\right)$, from the set of scattered field data observed on S. This is a nonlinear and ill-posed problem and is treated within the CSI [20] framework. Accordingly, the contrast sources are defined as

(6) $$w^{l}\left(r\right)=\chi \left(r\right)\;u^{l}\left(r\right),\;\;\;\;\;\;\;r\in D,\;\;\;\;\;\;\;l=1,\ldots ,L.$$

Then, the system of integral Equations (4) and (5) can be rewritten compactly as

(7) $$u^{l}=u_{b}^{l}+G^{D}w^{l},\;\;\;\;\;\;\;\;\;\;$$

(8) $$u_{s}^{l}=G^{S}w^{l},\;\;\;\;\;\;\;\;\;\;\;\;\;\;\;\;\;\;\;\;\;$$

where $G^{D}$ and $G^{S}$ are the integral operators defined by

(9) $$\left(G^{D,S}\psi \right)\left(r\right)=\iint_{D}G_{b}\left(r;r^{\prime }\right){k_{b}}^{2}\left(r^{\prime }\right)\psi \left(r^{\prime }\right)dr^{\prime },\;\;\;r\in D,S.$$

Following the standard CSI formulation, the reconstruction is cast as the minimization of a functional F that combines the residuals in the object and data equations over all illuminations. For the present formulation, one can write

(10) $$F=\frac{\sum_{l}{\left\|u_{s}^{l}-G^{S}w^{l}\right\|}_{S}^{2}}{\sum_{l}{\left\|u_{s}^{l}\right\|}_{S}^{2}}+\frac{\sum_{l}{\left\|\chi u_{b}^{l}-w^{l}+\chi G^{D}w^{l}\right\|}_{D}^{2}}{\sum_{l}{\left\|\chi u_{b}^{l}\right\|}_{D}^{2}}$$

Here, $∥\cdot {∥}_{S}$ and $∥\cdot {∥}_{D}$ denote the $L^{2}$-norms over the measurement curve S and reconstruction domain D, respectively. The CSI algorithm alternately updates the contrast sources $w^{l}$ and the contrast function χ so as to minimize the functional F. The process is stopped when the cost functional converges within a prescribed tolerance or when a predetermined number of iterations is reached. The explicit expressions of the iterative process for the original CSI method can be found in [20]. In practical implementations of CSI, the reconstruction domain D is partitioned into $N_{D}$ sufficiently small cells, within each of which the constitutive parameters and the electric field are assumed to be constant. Under this discretization, the operators in (9) can be approximated as

(11) $$\left(G^{D,S}\psi \right)\left(r\right)\approx \sum_{d=1}^{N_{D}}\nu_{d}(r){k_{b}}^{2}\left(r_{d}\right)\psi \left(r_{d}\right),\;\;\;r\in D,S,$$

where $r_{d}=(\rho_{d}$,$\;\phi_{d}$) is the position vector of the center of each cell d and $\nu_{d}(r)=\iint_{cell\;d}G_{b}\left(r;r^{\prime }\right)dr^{\prime }$. Note that rapid evaluation of the background fields $u_{b}^{l}$ and the Green’s function $G_{b}$ in the multilayer structure plays an important role in the practical efficiency of this procedure. The background fields $u_{b}^{l}$ can be computed efficiently via the recursive eigenfunction-expansion approach introduced in our previous work [24]. In the present study, this recursive formulation is extended to the computation of the Green’s function of the multilayer background and to the evaluation of the cell integrals appearing in (11), which are required in the CSI iterations. The details are presented in the following section.

## 3. Computation of the Green’s Function via Recursive EE-NCS

Below, in Section 3.1, we summarize the previously proposed EE-NCS procedure for the computation of the 2-D Green’s function of the multilayer background, $G_{b}\left(r;r^{\prime }\right)$ [23]. Then, in Section 3.2, by using the relations given in Section 3.1, we derive recursive expressions for a faster solution.

### 3.1. Summary of the Non-Recursive EE-NCS

The Green’s function $G_{b}\left(r;r^{\prime }\right)$ can be interpreted as the total field generated by a z-directed line source and decomposed into the functions $G_{m}\left(r;r^{\prime }\right)$, m=1,2,…,M, where r denotes the observation point located in the mth layer and the source point $r^{\prime }$ is an arbitrary point on the transverse plane. These functions satisfy the Helmholtz equation

(12) $$∆G_{m}\left(r;r^{\prime }\right)+{k_{m}}^{2}G_{m}\left(r;r^{\prime }\right)=-\delta \left(r-r^{\prime }\right).$$

subject to the continuity conditions

(13) $$G_{m-1}=G_{m}\;on\;\Gamma_{m}\;\;\;\;m=1,2,\ldots ,M$$

and

(14) $$\frac{\partial G_{m-1}}{\partial \rho }=\frac{\partial G_{m}}{\partial \rho }\;on\;\Gamma_{m}\;\;\;\;\;\;m=1,2,\ldots ,M.$$

When the source point also lies in the mth layer, $G_{m}\left(r;r^{\prime }\right)$ can be separated as $G_{m}\left(r;r^{\prime }\right)=G_{m}^{i}\left(r;r^{\prime }\right)+G_{m}^{s}\left(r;r^{\prime }\right)$ where

(15) $$G_{m}^{i}\left(r;r^{\prime }\right)=\frac{i}{4}H_{0}^{\left(1\right)}\left(k_{m}\left|r-r^{\prime }\right|\right)\;\;\;\;\;$$

denotes the Green’s function of the homogeneous medium characterized by the constitutive parameters of the mth layer. Accordingly, $G_{m}(r;r^{\prime })$ can be expressed as

(16) $$G_{m}\left(\rho ,\phi ;\rho \prime ,\phi \prime \right)\approx \delta_{m\tau }G_{m}^{i}\left(\rho ,\phi ;\rho^{\prime },\phi^{\prime }\right)+\sum_{n=-N}^{N}\left(a_{n}^{\left(m\right)}S_{n}^{1}\left(k_{m}\rho \right)+b_{n}^{\left(m\right)}S_{n}^{2}\left(k_{m}\rho \right)\right)e^{in\phi }$$

when the observation point $r=\left(\rho ,\phi \right)$ is in layer m and the source point $r^{\prime }=\left(\rho^{\prime },\phi^{\prime }\right)$ is in layer $\tau \in \left[0,M\right].$ In this representation, $\delta_{m\tau }$ is the Kronecker delta, equal to unity when m=τ and zero otherwise, while $S_{n}^{1}$ and $S_{n}^{2}$ are the $n^{th}$ order Bessel function $J_{n}$ and Hankel function of the first kind $H_{n}^{\left(1\right)}$ respectively. Note that (16) is theoretically an infinite series. Although the series in (16) is theoretically infinite, it may be truncated at a sufficiently large value N. The coefficients $a_{n}^{\left(m\right)}$ and $b_{n}^{\left(m\right)}$ are unknown quantities to be determined. Similarly, the radial derivative of $G_{m}$ may be represented as

(17) $$\frac{\partial G_{m}\left(\rho ,\phi ;\rho \prime ,\phi \prime \right)}{\partial \rho }=\delta_{ms}\frac{\partial G_{m}^{i}\left(\rho ,\phi ;\rho \prime ,\phi \prime \right)}{\partial \rho }+\sum_{n=-N}^{N}\left(a_{n}^{\left(m\right)}S_{n}^{3}\left(k_{m}\rho \right)+b_{n}^{\left(m\right)}S_{n}^{4}\left(k_{m}\rho \right)\right)e^{in\phi }.$$

where $S_{n}^{3}$ and $S_{n}^{4}$ denote the radial derivatives $\partial J_{n}/\partial \rho$ and $\partial H_{n}^{\left(1\right)}/\partial \rho$, respectively. These derivatives, together with $\partial G_{m}^{i}/\partial \rho$, are evaluated analytically. In the EE-NCS formulation (see [23] for details), substituting (16) and (17) into the continuity conditions on $\Gamma_{m}$ leads to the linear system

(18) $$\left[\begin{array}{c} \begin{array}{cc} \begin{array}{cc} Z_{m}^{1,\left(m-1\right)} & Z_{m}^{2,\left(m-1\right)} \end{array} & -\begin{array}{cc} Z_{m}^{1,\left(m\right)} & -Z_{m}^{2,\left(m\right)} \end{array} \end{array}\\ \begin{array}{cc} \begin{array}{cc} Z_{m}^{3,\left(m-1\right)} & Z_{m}^{4,\left(m-1\right)} \end{array} & \begin{array}{cc} {-Z}_{m}^{3,\left(m\right)} & {-Z}_{m}^{4,\left(m\right)} \end{array} \end{array} \end{array}\right]\left[\begin{array}{c} A^{\left(m-1\right)}\\ B^{\left(m-1\right)}\\ \begin{array}{c} A^{\left(m\right)}\\ B^{\left(m\right)} \end{array} \end{array}\right]=\left[\begin{array}{c} L_{m}\\ L_{m}^{*} \end{array}\right]$$

on each $\Gamma_{m},\;m=1,2,\ldots ,M$. Here, $Z_{m}^{t,(j)},\;t=1,2,3,4$ are sub-matrices of size $\left(2N+1\right)\times \left(2N+1\right)$, explicitly given by

(19) $$Z_{m}^{t,(j)}=\left[\begin{array}{ccc} \begin{array}{cc} C_{2N,-N,m}^{t,(j)} & C_{2N-1,-N+1,m}^{t,(j)}\\ C_{2N-1,-N,m}^{t,(j)} & C_{2N-2,-N+1,m}^{t,(j)} \end{array} & \ldots & \begin{array}{c} C_{0,N,m}^{t,(j)}\\ C_{-1,N,m}^{t,(j)} \end{array}\\ \begin{array}{cc} \vdots & \;\;\;\;\;\;\;\;\;\;\vdots \end{array} & \ddots & \vdots \\ \begin{array}{cc} C_{0,-N,m}^{t,(j)} & C_{-1,-N+1,m}^{t,(j)} \end{array} & \ldots & C_{-2N,N,m}^{t,(j)} \end{array}\right]$$

The vectors $A^{\left(m-1\right)}$, $B^{\left(m-1\right)}$, $A^{\left(m\right)}$ and $B^{\left(m\right)}$ in (18) are column vectors of size 2N+1 whose elements are $a_{n}^{\left(m-1\right)}$, $b_{n}^{\left(m-1\right)}$, $a_{n}^{\left(m\right)}$ and $b_{n}^{\left(m\right)}$ respectively. Moreover, $L_{m}$ is a column vector of the same size with elements

(20) $$\delta_{m\tau }\langle \;{\left.G_{m}^{i}\left(\rho ,\phi ;\rho^{\prime },\phi^{\prime }\right)\right|}_{\rho =f_{m}\left(\phi \right)},\;e^{-ip\phi }\rangle -\delta_{(m-1)\tau }\langle {\left.G_{m-1}^{i}\left(\rho ,\phi ;\rho^{\prime },\phi^{\prime }\right)\right|}_{\rho =f_{m}\left(\phi \right)},\;e^{-ip\phi }\rangle$$

for p=−N,…,N. The inner product appearing in (20) is defined by $\langle g_{1},g_{2}\rangle =\frac{1}{2\pi }\int_{0}^{2\pi }g_{1}\left(\phi \right)\bar{g_{2}\left(\phi \right)}d\phi$ for ${g_{1},g}_{2}\in L^{2}\left[0,2\pi \right]$, where the overbar denotes complex conjugation. The elements of $L_{m}^{*}$, which has the same dimensions as $L_{m}$, can be obtained from (20) by replacing $G_{m}^{i}$ and $G_{m-1}^{i}$ with their corresponding radial derivatives.

Writing (18) for all interfaces together yields a larger linear system that may be written compactly in matrix form as Zx=y. Here, Z is a matrix of size $2M\left(2N+1\right)\times 2(M+1)\left(2N+1\right)$, and the unknown vector x is a column vector of size $2(M+1)\left(2N+1\right)$; both are given explicitly in [26]. y, which is a column vector of size $2M\left(2N+1\right)$, is written as

(21) $$y=\left[\begin{array}{c} L_{1}\\ L_{1}^{*}\\ L_{2}\\ L_{2}^{*}\\ \vdots \\ L_{M}\\ L_{M}^{*} \end{array}\right].$$

By applying the Sommerfeld radiation condition in the outermost layer and using the singular behavior of the Hankel functions at the origin, the system is transformed into a square system. In [23], this system was solved by conventional linear algebraic techniques.

### 3.2. Recursive Evaluation of Green’s Function Coefficients

Instead of solving the linear system Zx=y using conventional numerical solvers for the large almost block-diagonal matrix Z, a recursive procedure based on the Thomas algorithm is employed. The main idea is to start from the innermost interface and proceed recursively toward the outermost interface. For this purpose, a modified relation $R_{m}$ associated with the interface $\Gamma_{m}$ is obtained by eliminating the coefficient vectors of the layers interior to $\Gamma_{m}$. This relation contains only the coefficient vectors of the layer immediately outside $\Gamma_{m}$. In general, it can be written as $R_{m}:\;{\tilde{Z}}_{m}^{A}A^{\left(m-1\right)}+{\tilde{Z}}_{m}^{B}B^{\left(m-1\right)}={\tilde{L}}_{m},m=1,2,\ldots ,M.$ Here, ${\tilde{Z}}_{m}^{A}$ and ${\tilde{Z}}_{m}^{B}$ are modified matrices, whereas ${\tilde{L}}_{m}$ is a modified vector; all are computed recursively.

**Figure 2:**
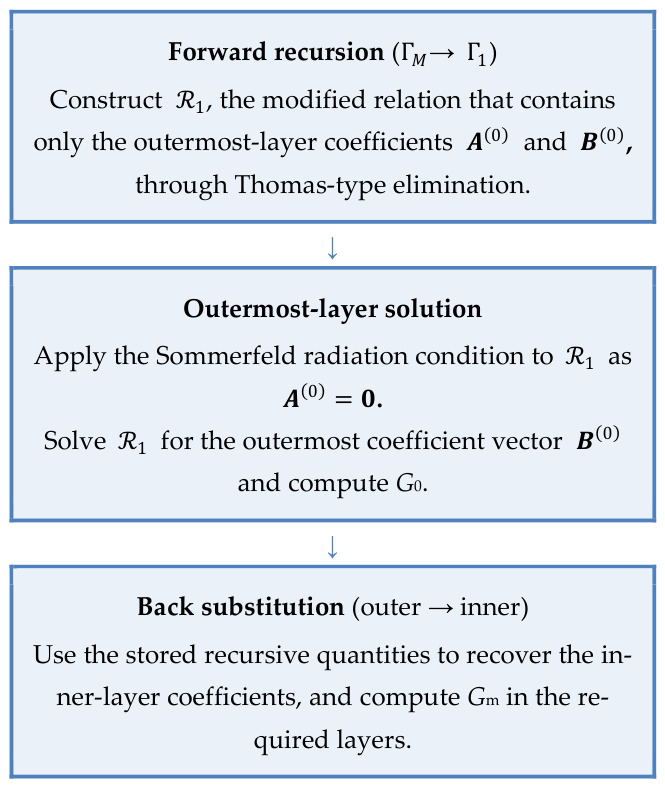
Main steps of the procedure for computing the background Green’s function.

At the end of this recursive process, $R_{1}$ is reached. The Sommerfeld radiation condition is then applied as $A^{\left(0\right)}=0$, and $B^{\left(0\right)}$ is obtained from $R_{1}$. Then, $G_{0}$ is computed from the corresponding expansion. Finally, the coefficient vectors of the inner layers are recovered by back substitution, which enables the computation of $G_{m}$ in the required layer. The main steps of this procedure are summarized in Figure 2, and the details are given below.

**Figure 3:**
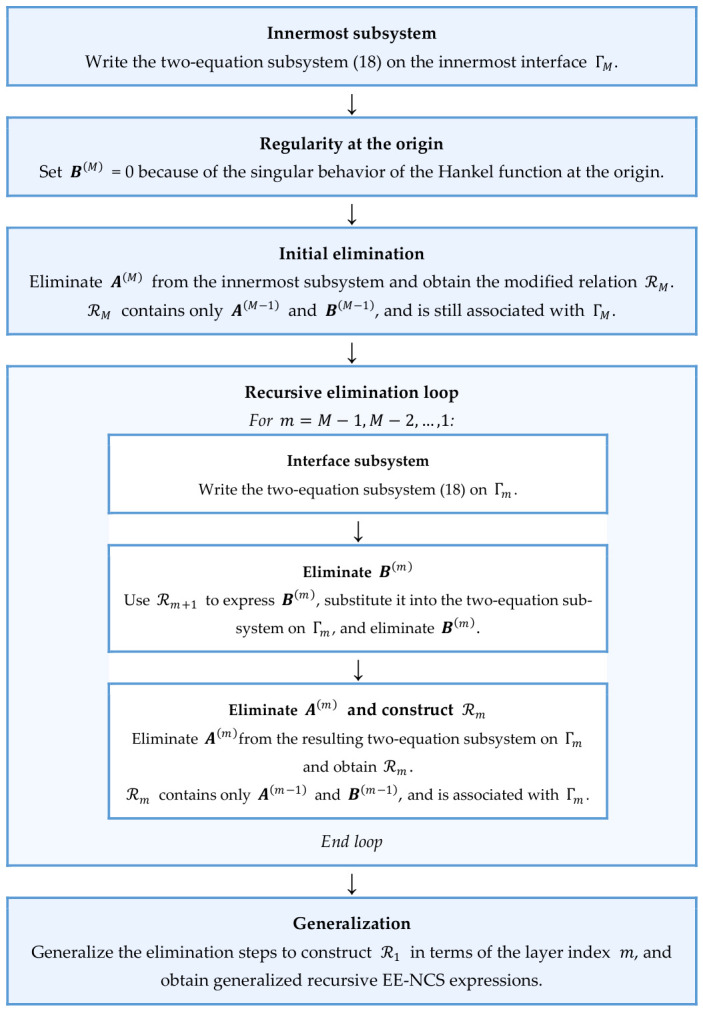
Thomas-type recursive elimination procedure used to construct the final modified relation $R_{1}$.

*Forward recursion*: The forward-recursion stage aims to construct the final modified relation $R_{1}$, which involves only the outermost-region coefficient vectors. This is achieved by successively eliminating the inner-layer coefficients through a Thomas-type procedure. The corresponding workflow is shown in Figure 3, and the details are presented below.

Consider first the subsystem corresponding to the innermost interface $\Gamma_{M}$

(22) $$Z_{M}^{1,\left(M-1\right)}A^{\left(M-1\right)}+Z_{M}^{2,\left(M-1\right)}B^{\left(M-1\right)}-Z_{M}^{1,\left(M\right)}A^{\left(M\right)}=L_{M}$$

(23) $$Z_{M}^{3,\left(M-1\right)}A^{\left(M-1\right)}+Z_{M}^{4,\left(M-1\right)}B^{\left(M-1\right)}-Z_{M}^{3,\left(M\right)}A^{\left(M\right)}=L_{M}^{*}$$

Because of the singular behavior of the Hankel function at the origin, the coefficient vector $B^{\left(M\right)}$ vanishes and hence does not exist in (22) and (23). By left-multiplying (22) by $Z_{M}^{3,\left(M\right)}{\left(Z_{M}^{1,\left(M\right)}\right)}^{-1}$ and subtracting (23), $A^{\left(M\right)}$ is eliminated, yielding the following modified relation, $R_{M}$, involving only the coefficients of layer M−1:

(24) $$R_{M}:\;\;\;{\tilde{Z}}_{M}^{A}A^{\left(M-1\right)}+{\tilde{Z}}_{M}^{B}B^{\left(M-1\right)}={\tilde{L}}_{M}$$

where

(25) $${\tilde{Z}}_{M}^{A}=Z_{M}^{3,\left(M\right)}{\left(Z_{M}^{1,\left(M\right)}\right)}^{-1}Z_{M}^{1,\left(M-1\right)}-Z_{M}^{3,\left(M-1\right)}$$

(26) $${\tilde{Z}}_{M}^{B}=Z_{M}^{3,\left(M\right)}{\left(Z_{M}^{1,\left(M\right)}\right)}^{-1}Z_{M}^{2,\left(M-1\right)}-Z_{M}^{4,\left(M-1\right)}$$

(27) $${\tilde{L}}_{M}=Z_{M}^{3,\left(M\right)}{\left(Z_{M}^{1,\left(M\right)}\right)}^{-1}L_{M}-L_{M}^{*}.$$

Using (24), the coefficient vector $B^{\left(M-1\right)}$ can be eliminated from the subsystem (18) associated with the interface $\Gamma_{M-1}$, that is, for m=M−1. To this end, (24) is left-multiplied separately by ${Z_{M-1}^{2,\left(M-1\right)}\left({\tilde{Z}}_{M}^{B}\right)}^{-1}$ and ${Z_{M-1}^{4,\left(M-1\right)}\left({\tilde{Z}}_{M}^{B}\right)}^{-1}$, and the resulting expressions are added to the first and second rows of (18), respectively. Consequently, one obtains

(28) $$Z_{M-1}^{1,\left(M-2\right)}A^{\left(M-2\right)}+Z_{M-1}^{2,\left(M-2\right)}B^{\left(M-2\right)}+\Psi_{M-1}^{1}A^{\left(M-1\right)}=\Phi_{M-1}^{1}$$

(29) $$Z_{M-1}^{3,\left(M-2\right)}A^{\left(M-2\right)}+Z_{M-1}^{4,\left(M-2\right)}B^{\left(M-2\right)}+\Psi_{M-1}^{2}A^{\left(M-1\right)}=\Phi_{M-1}^{2}$$

where

(30) $$\Psi_{M-1}^{1}=Z_{M-1}^{2,\left(M-1\right)}{\left({\tilde{Z}}_{M}^{B}\right)}^{-1}{\tilde{Z}}_{M}^{A}-Z_{M-1}^{1,\left(M-1\right)}$$

(31) $$\Psi_{M-1}^{2}=Z_{M-1}^{4,\left(M-1\right)}{\left({\tilde{Z}}_{M}^{B}\right)}^{-1}{\tilde{Z}}_{M}^{A}-Z_{M-1}^{3,\left(M-1\right)}$$

(32) $$\Phi_{M-1}^{1}=Z_{M-1}^{2,\left(M-1\right)}{\left({\tilde{Z}}_{M}^{B}\right)}^{-1}{\tilde{L}}_{M}+L_{M-1}$$

(33) $$\Phi_{M-1}^{2}=Z_{M-1}^{4,\left(M-1\right)}{\left({\tilde{Z}}_{M}^{B}\right)}^{-1}{\tilde{L}}_{M}+L_{M-1}^{*}$$

Next, in order to eliminate $A^{\left(M-1\right)}$ from (28) and (29), (28) is left-multiplied by $-\Psi_{M-1}^{2}{\left(\Psi_{M-1}^{1}\right)}^{-1}$ and added to (29). This yields

(34) $$R_{M-1}:\;\;\;{\tilde{Z}}_{M-1}^{A}A^{\left(M-2\right)}+{\tilde{Z}}_{M-1}^{B}B^{\left(M-2\right)}={\tilde{L}}_{M-1}$$

where

(35) $${\tilde{Z}}_{M-1}^{A}=\Psi_{M-1}^{2}{\left(\Psi_{M-1}^{1}\right)}^{-1}Z_{M-1}^{1,\left(M-2\right)}-Z_{M-1}^{3,\left(M-2\right)}$$

(36) $${\tilde{Z}}_{M-1}^{B}=\Psi_{M-1}^{2}{\left(\Psi_{M-1}^{1}\right)}^{-1}Z_{M-1}^{2,\left(M-2\right)}-Z_{M-1}^{4,\left(M-2\right)}$$

(37) $${\tilde{L}}_{M-1}=\Psi_{M-1}^{2}{\left(\Psi_{M-1}^{1}\right)}^{-1}\Phi_{M-1}^{1}-\Phi_{M-1}^{2}.$$

Thus, the coefficient vectors $B^{\left(M-1\right)}$ and $A^{\left(M-1\right)}$ are eliminated from the subsystem associated with $\Gamma_{M-1}$, yielding the modified relation $R_{M-1}$ in (34). Similarly, this relation can be used together with the subsystem on $\Gamma_{M-2}$ to eliminate the unknowns $B^{\left(M-2\right)}$ and $A^{\left(M-2\right)}$. This leads to a new modified relation, denoted by $R_{M-2}$, which has the same form as (34) and is written in terms of the coefficients of the next outer layer, namely $B^{\left(M-3\right)}$ and $A^{\left(M-3\right)}$. The coefficient matrices and the right-hand-side vector of this new relation are obtained in a form analogous to (35)–(37). Repeating this procedure successively for the boundaries $\Gamma_{M-3},\Gamma_{M-4},\ldots ,\Gamma_{1}$, one finally obtains the modified relation $R_{1}$ at the outermost interface as

(38) $${R_{1}:\;\;\;\tilde{Z}}_{1}^{A}A^{\left(0\right)}+{\tilde{Z}}_{1}^{B}B^{\left(0\right)}={\tilde{L}}_{1}$$

where

(39) $${\tilde{Z}}_{1}^{A}=\Psi_{1}^{2}{\left(\Psi_{1}^{1}\right)}^{-1}Z_{1}^{1,\left(0\right)}-Z_{1}^{3,\left(0\right)}$$

(40) $${\tilde{Z}}_{1}^{B}=\Psi_{1}^{2}{\left(\Psi_{1}^{1}\right)}^{-1}Z_{1}^{2,\left(0\right)}-Z_{1}^{4,\left(0\right)}$$

(41) $$\Psi_{1}^{1}=Z_{1}^{2,\left(1\right)}{\left({\tilde{Z}}_{2}^{B}\right)}^{-1}{\tilde{Z}}_{2}^{A}-Z_{1}^{1,\left(1\right)}$$

(42) $$\Psi_{1}^{2}=Z_{1}^{4,\left(1\right)}{\left({\tilde{Z}}_{2}^{B}\right)}^{-1}{\tilde{Z}}_{2}^{A}-Z_{1}^{3,\left(1\right)}$$

(43) $${\tilde{L}}_{1}=\Psi_{1}^{2}{\left(\Psi_{1}^{1}\right)}^{-1}\Phi_{1}^{1}-\Phi_{1}^{2}$$

(44) $$\Phi_{1}^{1}=Z_{1}^{2,\left(1\right)}{\left({\tilde{Z}}_{2}^{B}\right)}^{-1}{\tilde{L}}_{2}+L_{1}$$

(45) $$\Phi_{1}^{2}=Z_{1}^{4,\left(1\right)}{\left({\tilde{Z}}_{2}^{B}\right)}^{-1}{\tilde{L}}_{2}+L_{1}^{*}$$

The elimination steps described above can be generalized in terms of the layer index m. This yields the following recursive EE-NCS expressions for m=M,M−1,…,1. Starting from the initial values in (46)–(48) and applying the update relations in (49)–(54) successively, one obtains ${\tilde{Z}}_{1}^{A}$, ${\tilde{Z}}_{1}^{B}$, and ${\tilde{L}}_{1}$, which define the final modified relation $R_{1}$ given in (38).

(46) $${\tilde{Z}}_{M+1}^{A}=0,$$

(47) $${\tilde{Z}}_{M+1}^{B}=I,$$

(48) $${\tilde{L}}_{M+1}=0,$$

(49) $$\Psi_{m}^{j}=Z_{m}^{2j,\left(m\right)}{\left({\tilde{Z}}_{m+1}^{B}\right)}^{-1}{\tilde{Z}}_{m+1}^{A}-Z_{m}^{2j-1,\left(m\right)},\;j=1,2,$$

(50) $$\Phi_{m}^{1}=Z_{m}^{2,\left(m\right)}{\left({\tilde{Z}}_{m+1}^{B}\right)}^{-1}{\tilde{L}}_{m+1}+L_{m},$$

(51) $$\Phi_{m}^{2}=Z_{m}^{4,\left(m\right)}{\left({\tilde{Z}}_{m+1}^{B}\right)}^{-1}{\tilde{L}}_{m+1}+L_{m}^{*},$$

(52) $${\tilde{Z}}_{m}^{A}=\Psi_{m}^{2}{\left(\Psi_{m}^{1}\right)}^{-1}Z_{m}^{1,\left(m-1\right)}-Z_{m}^{3,\left(m-1\right)},$$

(53) $${\tilde{Z}}_{m}^{B}=\Psi_{m}^{2}{\left(\Psi_{m}^{1}\right)}^{-1}Z_{m}^{2,\left(m-1\right)}-Z_{m}^{4,\left(m-1\right)},$$

(54) $${\tilde{L}}_{m}=\Psi_{m}^{2}{\left(\Psi_{m}^{1}\right)}^{-1}\Phi_{m}^{1}-\Phi_{m}^{2}.$$

*Outermost-layer solution*: The Sommerfeld radiation condition implies $A^{\left(0\right)}=0$. Hence, the final modified relation $R_{1}$ reduces to ${\tilde{Z}}_{1}^{B}B^{\left(0\right)}={\tilde{L}}_{1}$, and $B^{\left(0\right)}$ can be computed from this relation. Consequently, $G_{0}$ can be computed by using (16) for the obtained $B^{\left(0\right)}$ and $A^{\left(0\right)}=0$.

*Back substitution*: When the observation point is in the inner layers, the required unknown coefficients can be obtained by back substitution. More precisely, by applying m=M−1 transformation in (28) and (24),

(55) $$A^{\left(m\right)}={\left(\Psi_{m}^{1}\right)}^{-1}\left(\Phi_{m}^{1}-Z_{m}^{1,\left(m-1\right)}A^{\left(m-1\right)}-Z_{m}^{2,\left(m-1\right)}B^{\left(m-1\right)}\right),$$

(56) $$B^{\left(m\right)}=\left\{\begin{array}{c} 0,\;\;\;\;\;\;\;\;\;\;\;\;\;\;\;\;\;\;\;\;\;\;\;\;\;\;\;\;\;\;\;\;\;\;\;\;m=M\;\\ {\left({\tilde{Z}}_{m+1}^{B}\right)}^{-1}\left({\tilde{L}}_{m+1}-{\tilde{Z}}_{m+1}^{A}A^{\left(m\right)}\right),\;m=1,2,\ldots M-1 \end{array}\right.$$

can be computed for m=1, 2,…,M, which enables direct computation of $G_{m}$ via (16).

As a final note, when the innermost layer is a perfect electric conductor (PEC), the boundary condition on $\Gamma_{M}$ is imposed as $G_{M}=0$, instead of the continuity conditions used above. In this case, the recursive procedure starts with

(57) $$Z_{M}^{1,\left(M-1\right)}A^{\left(M-1\right)}+Z_{M}^{2,\left(M-1\right)}B^{\left(M-1\right)}=L_{M}$$

instead of (22) and (23). Accordingly, the initial values in (46), (47), and (48) become ${\tilde{Z}}_{M}^{A}=Z_{M}^{1,\left(M-1\right)}$, ${\tilde{Z}}_{M}^{B}=Z_{M}^{2,\left(M-1\right)}$, and ${\tilde{L}}_{M}=L_{M}$, respectively. In addition, the index m in (49)–(54) starts from M−1 rather than M. Apart from these modifications, the recursive relations in (46)–(54) remain unchanged.

### 3.3. Recursive Evaluation of the Cell Integral of Green’s Function Coefficients

We define the integral of $G_{m}$ over each cell *d*, which is required for the implementation of CSI (see (11)), as

(58) $$\nu_{m,d}\left(r\right)=\iint_{cell\;d}G_{m}\left(r;r^{\prime }\right)dr^{\prime }$$

for each layer m,m=0,1,2,…,M. Then, integrating both sides of (12) over cell *d*, one can obtain

(59) $$∆\nu_{m,d}\left(r\right)+k_{m}^{2}\nu_{m,d}\left(r\right)=-\iint_{cell\;d}\delta \left(r-r^{\prime }\right)dr^{\prime }.$$

The procedure given for the computation of $G_{m}$ in Section 3.2 above can be applied for the computation of $\nu_{m,d}$. In this case, the only difference lies in the elements of $L_{m}$ given in (20) and, consequently, in $L_{m}^{*}$, as detailed in [27]. Then, the elements of $\left(2N+1\right)$-sized vectors $L_{m}$ and $L_{M}^{*}$ become

(60) $$\delta_{m\tau }\langle \;{\left.\nu_{m,d}^{i}\left(\rho ,\phi \right)\right|}_{\rho =f_{m}\left(\phi \right)},\;e^{-ip\phi }\rangle -\delta_{\left(m-1\right)\tau }\langle \;{\left.\nu_{\left(m-1\right),d}^{i}\left(\rho ,\phi \right)\right|}_{\rho =f_{m}\left(\phi \right)},\;e^{-ip\phi }\rangle$$

and

(61) $$\delta_{m\tau }\langle \;{\left.\frac{\partial \nu_{m,d}^{i}\left(\rho ,\phi \right)}{\partial \rho }\right|}_{\rho =f_{m}\left(\phi \right)},\;e^{-ip\phi }\rangle -\delta_{\left(m-1\right)\tau }\langle {\left.\frac{\partial \nu_{\left(m-1\right),d}^{i}\left(\rho ,\phi \right)}{\partial \rho }\right|}_{\rho =f_{m}\left(\phi \right)},\;e^{-ip\phi }\rangle$$

respectively, where p=−N,…,N,

(62) $$\nu_{m,d}^{i}\left(r\right)=\left\{\begin{array}{c} \frac{-i}{2k_{m}^{2}}\left(\pi k_{m}\xi_{d}H_{0}^{\left(1\right)}\left(k_{m}\xi_{d}\right)+2i\right),\;\;\;r\in \mathrm{cell}\;d\;\;\;\\ \frac{-i\pi a}{2k_{m}}J_{1}\left(k_{m}\xi_{d}\right)H_{0}^{\left(1\right)}\left(k_{m}\left|r-r_{d}\right|\right),\;\;\;r\notin \mathrm{cell}\;d \end{array}\right.$$

and

(63) $$\frac{\partial \nu_{m,d}^{i}\left(r\right)}{\partial \rho }=\frac{-i\pi a\left(\rho -\rho_{d}\cos\left(\phi_{d}-\phi \right)\right)}{2\left|r-r_{d}\right|}\times J_{1}\left(k_{m}\xi_{d}\right)H_{-1}^{\left(1\right)}\left(k_{m}\left|r-r_{d}\right|\right).\;$$

It should be noted that, when r∈ cell d (60) vanishes for p≠0, whereas (61) vanishes for all values of p. In (62) and (63), $\xi_{d}$ denotes the radius of the circular cell having the same cross-sectional area as cell d.

## 4. Numerical Results

In order to assess the performance of the proposed imaging approach, numerical simulations are carried out for dielectric objects embedded in various multilayer cylindrical structures. Unless otherwise specified, single-frequency TM_z_ polarized illuminations at f=3 GHz are considered in the presented examples. A total of L=20 line sources are uniformly distributed on a circle of radius 14 cm enclosing the structure. For each illumination, the total field $u^{l}$ is computed at 100 equidistant observation points located on a circle S of radius 13 cm by solving the forward scattering problem using a Method of Moments (MoM) formulation. To evaluate robustness, a random term $\eta \left|u^{l}\right|e^{i2\pi r_{d}}$ is added to the synthetic total field data, where η = 0.0316 is the noise level and *r_d_* is a uniformly distributed random variable between 0 and 1. The corresponding signal to noise ratio is then $SNR=-20\log_{10}\eta ≅30$ dB. Then, the scattered field $u_{s}^{l}$ on S is obtained by $u_{s}^{l}=u^{l}-u_{b}^{l}$, where $u_{b}^{l}$ is the background field. Note that, since $u^{l}$ is computed via MoM whereas $u_{b}^{l}$ is generated via the method proposed in this paper, the mismatch between the two forward models can be regarded as an extra noise source in the inversion process.

The CSI algorithm is executed with a maximum of 500 iterations. The iterative process is terminated when the difference between two successive error values falls below $10^{-9}$. The reconstruction domain D is discretized into cells of side length $\lambda_{b}/12$, where $\lambda_{b}$ denotes the minimum wavelength in the background medium. Unless otherwise specified, a positivity constraint on the contrast is enforced throughout the CSI procedure. In all simulations, the truncation number in the EE-NCS formulation is set to N=40. All simulations were carried out on a desktop computer equipped with an AMD Ryzen 7 processor and 32 GB of RAM, without GPU acceleration. The forward MoM reference solver, the proposed recursive EE-NCS routines, and the CSI were all implemented in MATLAB R2025b. To complement the visual assessment of the reconstructed images, the normalized reconstruction error (NRE), defined as the relative $L^{2}$-norm error over $\Omega_{cyl}$, is introduced for the relative permittivity and conductivity profiles as

(64) $${NRE}_{\varepsilon }=\frac{{∥{\hat{\varepsilon }}_{r}-\varepsilon_{r}∥}_{\Omega_{cyl}}}{{∥\varepsilon_{r}∥}_{\Omega_{cyl}}},{NRE}_{\sigma }=\frac{{∥\hat{\sigma }-\sigma ∥}_{\Omega_{cyl}}}{{∥\sigma ∥}_{\Omega_{cyl}}}.$$

Here, $\varepsilon_{r}$ and σ denote the exact relative permittivity and conductivity profiles, respectively, whereas ${\hat{\varepsilon }}_{r}$ and $\hat{\sigma }$ denote their reconstructed counterparts. The region $\Omega_{cyl}$ denotes the cross-sectional region occupied by the entire multilayer cylinder.

**Figure 4:**
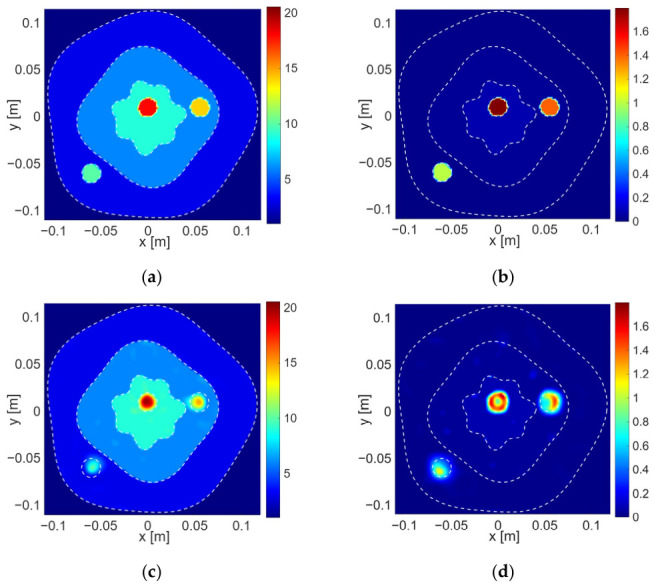

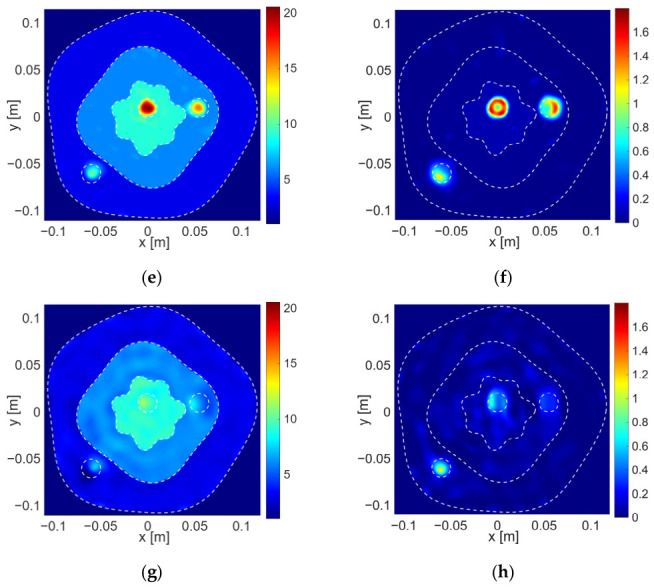
Three circular objects embedded in a three-layer cylindrical structure. Exact (**a**) relative permittivity and (**b**) conductivity distributions. (**c**,**d**) Reconstructions using the proposed method. (**e**,**f**) Reconstructions using CSI, where $u_{b}^{\left(l\right)}$ and the required Green’s functions are computed via MoM. (**g**,**h**) Reconstructions using the standard CSI with free space as the background and the known multilayer profiles used as the initial estimate.

**Figure 5:**
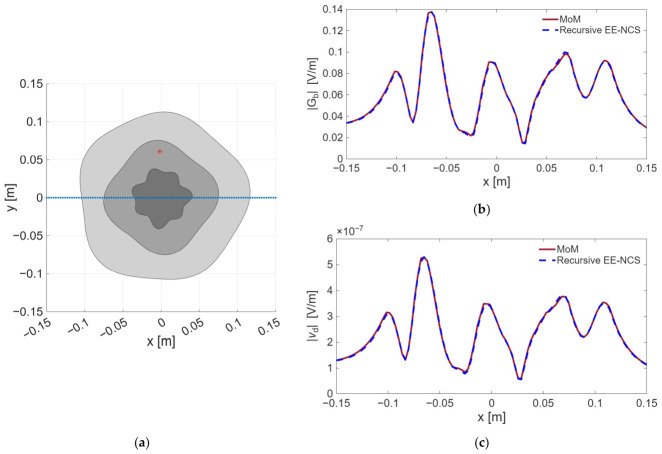
(**a**) Background medium of the first example with the source point (red asterisk) and observation points (blue dots) selected for the validation of (**b**) the Green’s function and (**c**) its cell-integrated counterpart (with integration performed over the discretization cell centered at the source location).

In the first example, three circular objects, each with a radius of 1 cm, are embedded in a three-layer cylindrical structure, as shown in Figure 4a,b, where the relative permittivity and conductivity distributions are depicted. The relative permittivities of the embedded objects are 10, 14, and 18, while their conductivities are 1.0 S/m, 1.4 S/m, and 1.8 S/m, respectively. The relative permittivities and conductivities of the layers, from the outermost to the innermost, are 3, 6, and 9, and 0.02 S/m, 0.04 S/m, and 0.08 S/m, respectively. Before examining the CSI results, let us first demonstrate the accuracy of the Green’s function and its cell integrals, computed using the proposed recursive formulation. For this purpose, the source and observation points are chosen as shown in Figure 5a, where the source point is marked by a red asterisk and the observation points by blue dots along a line intersecting all layers. The Green’s functions and their cell-integrated counterparts (with integration performed over the discretization cell centered at the source location) are compared with those computed by using MoM, confirming the accuracy of the recursive EE-NCS part of the proposed approach, as seen in Figure 5b,c. For the CSI reconstruction, the domain D is chosen as the region occupied by the entire multilayer cylinder and discretized into cells. The boundary of the domain D is depicted as a red curve in Figure 4. The reconstructed relative permittivity and conductivity profiles obtained using the proposed method are shown in Figure 4c,d, respectively. The corresponding reconstruction errors are ${NRE}_{\varepsilon }=0.13$ and ${NRE}_{\sigma }=0.45$. The results demonstrate that the method successfully recovers the spatial locations, sizes, and quantitative values of both permittivity and conductivity profiles. Although minor distortions are observed in the conductivity reconstruction, multiple targets are reliably characterized within the heterogeneous background. For comparison, the background field and the required Green’s functions are also computed using MoM instead of the proposed recursive approach. To this end, MoM is applied with the same discretization used in the inversion, and the corresponding reconstructions are presented in Figure 4e,f. For the reconstructions in Figure 4e,f, the inversion algorithm is still CSI and the multilayer host is again incorporated into the background model. The only difference is that the background field and the required Green’s functions are computed using MoM instead of the proposed recursive EE-NCS formulation. Thus, this comparison assesses the accuracy of the proposed background-field and Green’s-function computations within the same CSI framework. It is observed that both approaches yield nearly identical results; in the MoM-based case, the corresponding reconstruction errors are ${NRE}_{\varepsilon }=0.13$ and ${NRE}_{\sigma }=0.46$, which are almost the same as those obtained by the proposed method. However, the total computation times required to evaluate the background field $u_{b}^{l}$ for all illuminations and to compute all required Green’s functions are approximately 40.6 s and 88.2 s, respectively, whereas these times are reduced to 1.2 s and 12.1 s when the proposed recursive approach is employed. Each CSI iteration takes approximately 0.3 s for both approaches. It is observed that similar reconstruction results are obtained when the background field and the required Green’s functions are computed using MoM and when they are computed via the proposed method. Therefore, such comparisons are omitted in the subsequent numerical examples for brevity.

One might suggest applying the standard CSI algorithm in a direct manner to image the entire configuration, including the multilayer structure, by treating free space as the background and using the known permittivity and conductivity profiles of the multilayer structure as the initial estimate. In this case, as shown in Figure 4g,h, the embedded objects are reasonably well localized; however, both the permittivity and conductivity values are significantly underestimated. The corresponding reconstruction errors are ${NRE}_{\varepsilon }=1.68$ and ${NRE}_{\sigma }=0.80$, which are considerably larger than those obtained by the proposed method, namely ${NRE}_{\varepsilon }=0.13$ and ${NRE}_{\sigma }=0.45$. These results confirm that incorporating a priori information about the multilayer structure into the background, as in the proposed approach, substantially improves the performance of CSI. Note that all permittivity profiles are displayed using identical colorbars for a fair visual comparison. The upper bounds of the colorbars are set to the maximum values across all profiles. The same applies to the conductivity profiles.

**Figure 6:**
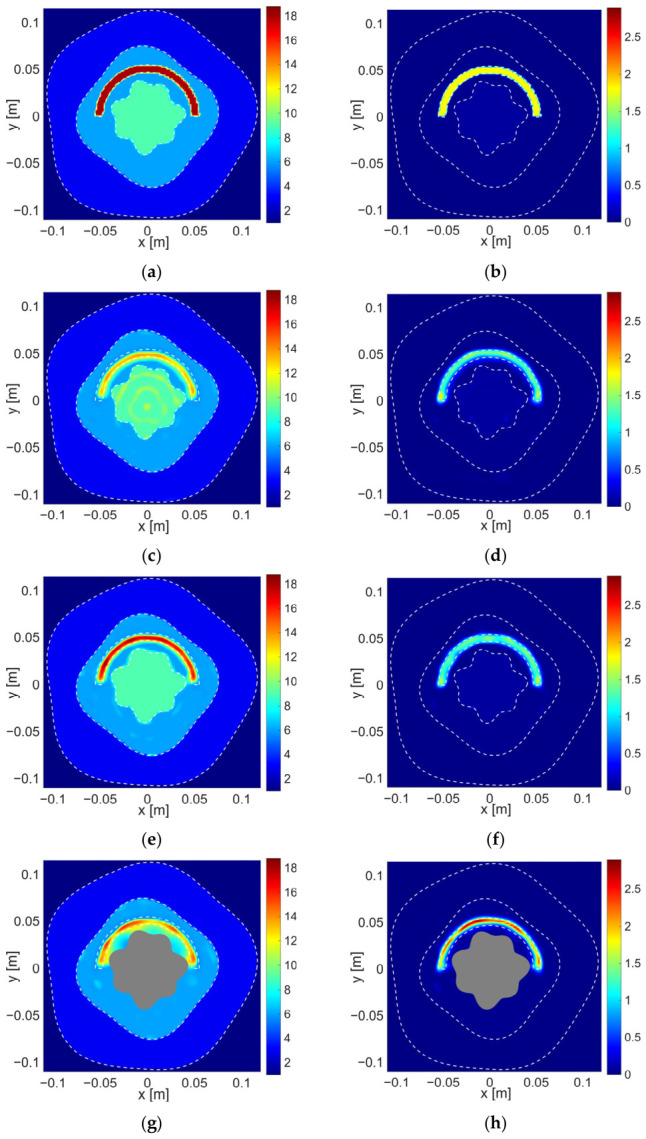
Arc-shaped object embedded in a three-layered cylindrical structure. Exact (**a**) relative permittivity and (**b**) conductivity distributions. (**c**,**d**) Reconstructions using the proposed method with the reconstruction domain D chosen as the entire cross-section. (**e**,**f**) Reconstructions with D restricted to the middle layer of the multilayer cylinder. (**g**,**h**) Reconstructions when the core is a PEC.

In the second example, instead of isolated circular objects, a thin arc-shaped object is embedded in the same multilayer cylindrical structure. The relative permittivity and conductivity of the object are 18 and 1.8 S/m, respectively (see Figure 6a,b). The reconstruction domain D is initially chosen as the region occupied by the entire multilayer cylinder and discretized into $N_{D}=9892$ cells, as in the previous example. As shown in Figure 6c,d, the arc-shaped object is well localized and its shape is accurately reconstructed. However, the reconstructed permittivity is approximately 14 instead of 18, and minor artifacts are observed in the core layer. The corresponding reconstruction errors are ${NRE}_{\varepsilon }=0.27$ and ${NRE}_{\sigma }=0.48$. Since the proposed method allows restricting the reconstruction domain, imaging is also performed for the same configuration by limiting D to the second (middle) layer of the cylinder. As shown in Figure 6e,f, this restriction improves the reconstructed permittivity values, and no significant artifacts are observed. The corresponding reconstruction errors are ${NRE}_{\varepsilon }=0.23$ and ${NRE}_{\sigma }=0.46$, which are slightly lower than those obtained when the entire multilayer cylinder is used as the reconstruction domain. As a final variation in this example, the dielectric core is replaced by a PEC, and the reconstructions shown in Figure 6g,h are obtained. A certain degradation in the reconstruction quality is observed. The corresponding reconstruction errors are ${NRE}_{\varepsilon }=0.32$ and ${NRE}_{\sigma }=0.46$. This degradation may be mainly attributed to the stronger multiple scattering and sharper field variations induced by the perfectly conducting boundary. These effects make the inverse scattering problem more challenging and may reduce the accuracy of the quantitative reconstruction. Nevertheless, the location and overall shape of the embedded object are still successfully recovered.

**Figure 7:**
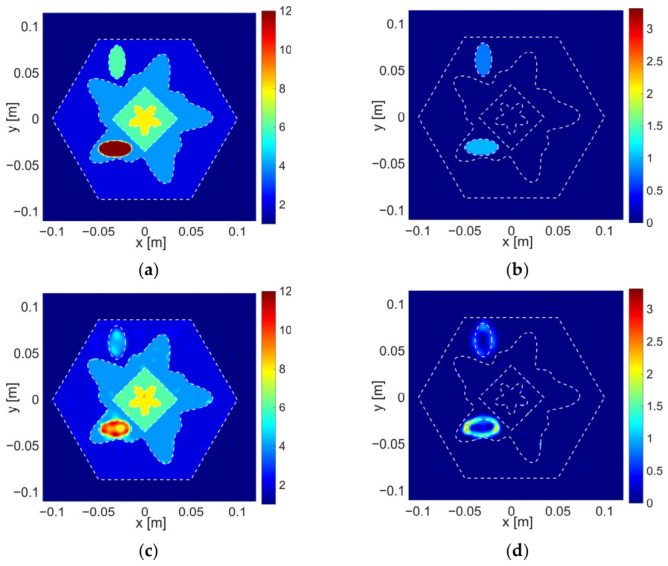
Two elliptical objects embedded in a four-layered cylinder with relatively complex boundaries. Exact (**a**) relative permittivity and (**b**) conductivity distributions. (**c**,**d**) Reconstructions using the proposed method at 5 GHz.

**Figure 8:**
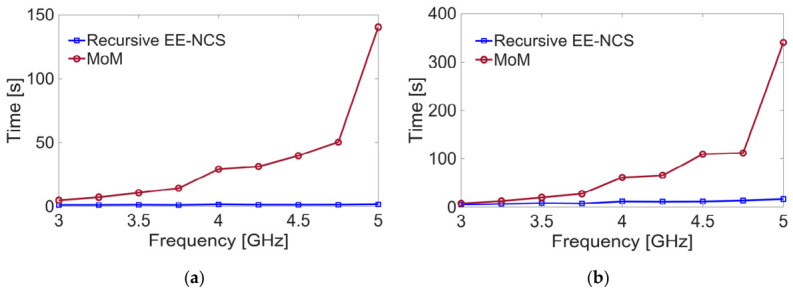
Comparison of the computational times required by MoM and the proposed approach to compute (**a**) the background field $u_{b}^{l}$ and (**b**) the background Green’s functions over the frequency range 3–5 GHz.

In the next example, two elliptical objects are embedded in a four-layer cylinder with relatively complex boundaries as shown in Figure 7a,b. The relative permittivities and conductivities of the layers, from the outermost to the innermost, are 2, 4, 6, 8 and 0.002 S/m, 0.004 S/m, 0.006 S/m, 0.08 S/m, respectively. The relative permittivities of the embedded objects are 6 and 12, while their conductivities are 0.8 S/m and 1.0 S/m, respectively. To examine the impact of the electrical size of the multilayer cylinder on computational time, the operating frequency is varied from 3 GHz to 5 GHz in steps of 250 MHz, and at each frequency the entire imaging procedure, including the computation of the background field and the background Green’s function, is performed separately. As the operating frequency increases, the number of cells ranges from 4494 to 12,446. The total computation times required to evaluate the background field $u_{b}^{l}$ for all illuminations and to compute the required Green’s functions are plotted as functions of frequency in Figure 8a,b, respectively. As the frequency (and hence the electrical size) increases, the computational advantage of the proposed method becomes more pronounced. On the other hand, the accuracy of the EE-NCS approach used for computing the background field and the background Green’s function may tend to degrade for electrically large multilayer cylinders, as discussed in [23]. In this context, the reconstruction results at 5 GHz, corresponding to the largest electrical size considered, are presented in Figure 7c,d. Even at this frequency, satisfactory results are obtained, although the conductivity values are not reconstructed with high accuracy. The corresponding reconstruction errors are ${NRE}_{\varepsilon }=0.17$ and ${NRE}_{\sigma }=1.00$, indicating that the degradation is much more pronounced in the conductivity reconstruction. In particular, the conductivity profile tends to be reconstructed in a ring-like form around the objects, rather than as a quantitatively accurate distribution. It should be noted that such inaccuracies are commonly observed in CSI-based reconstructions, even in free space, and similar behavior is also observed in this example when the background field and the required Green’s functions are computed using MoM.

**Figure 9:**
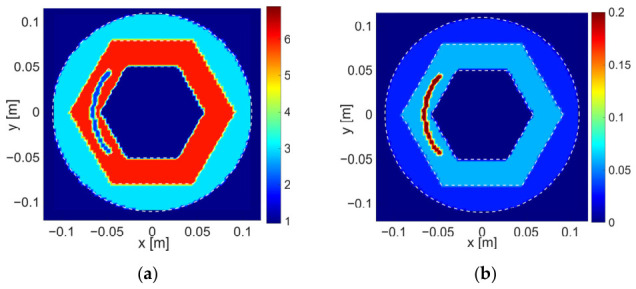

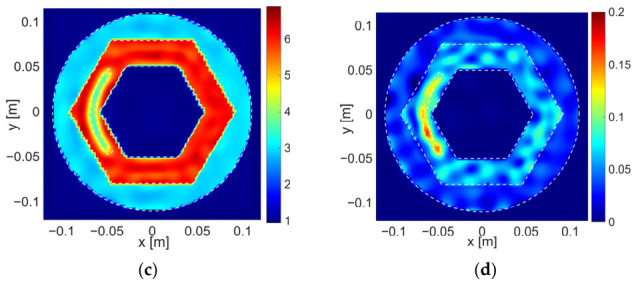
Arc-shaped object embedded in a three-layer cylindrical structure with a hollow core. The real part of the contrast with respect to the host layer is negative. Exact (**a**) relative permittivity and (**b**) conductivity distributions. (**c**,**d**) Reconstructions obtained without imposing the positivity constraint.

In the examples considered so far, both the real and imaginary parts of the contrast function associated with the object were positive. Assuming that this property is known a priori, the positivity constraint formulations given in [20] were incorporated into the CSI iterations. In this example, in order to assess the performance of the inversion without imposing the positivity constraint, an arc-shaped object is embedded in a three-layer cylindrical structure with a hollow (free-space) core, where the relative permittivities and conductivities of the layers, from the outermost to the innermost, are 3, 6, 1 and 0.03 S/m, 0.06 S/m, 0 S/m, respectively (see Figure 9a,b). The embedded object is assigned a relative permittivity of 2 and a conductivity of 0.2 S/m, resulting in a contrast whose real part is approximately −0.66 with respect to the host layer. In the absence of the positivity constraint, satisfactory reconstruction results in terms of shape and location are obtained, while the permittivity values are overestimated to a limited extent (see Figure 9c,d). The corresponding reconstruction errors are ${NRE}_{\varepsilon }=0.08$ and ${NRE}_{\sigma }=0.37$. This example also indicates that, when the positivity constraint is removed, the inversion becomes less constrained and may therefore be more sensitive to noise and modeling errors. This may explain the limited overestimation observed in the permittivity reconstruction for the negative-contrast case. Nevertheless, the relatively small value of ${NRE}_{\varepsilon }$ and the successful recovery of the object location show that the proposed method can still provide satisfactory reconstructions without the positivity constraint in this example.

**Figure 10:**
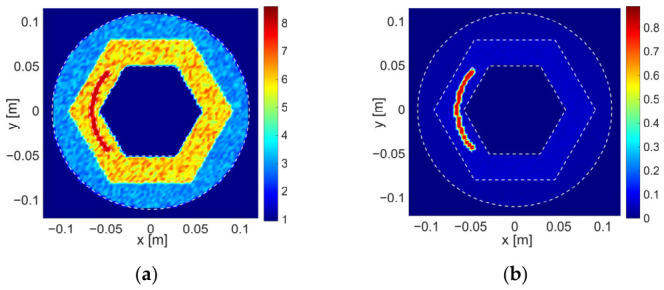

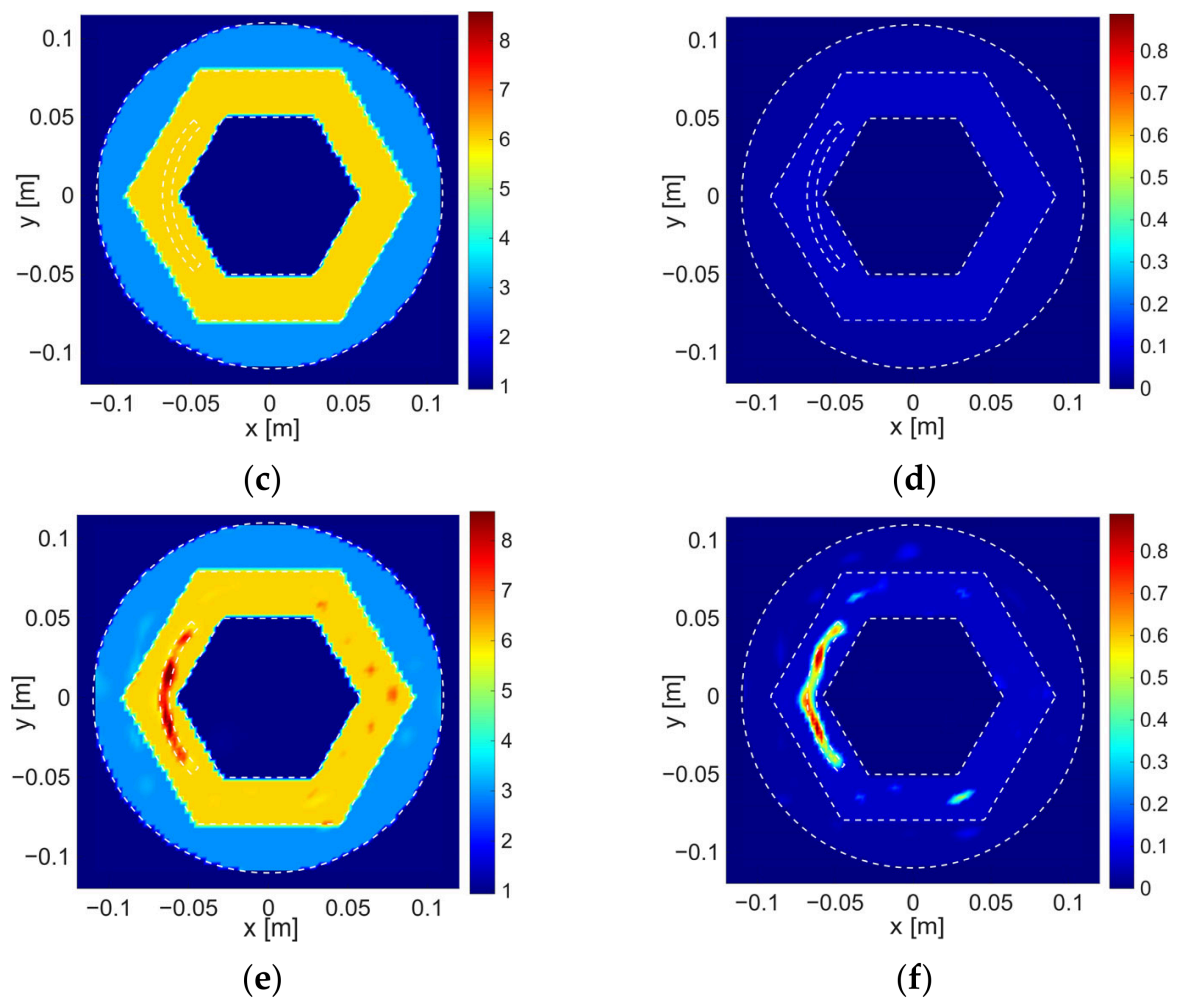
Arc-shaped object embedded in a three-layer cylindrical structure with a hollow core, where the background medium exhibits inhomogeneous variations around reference values. Exact (**a**) relative permittivity and (**b**) conductivity distributions. (**c**,**d**) Assumed background permittivity and conductivity profiles used in the imaging process. (**e**,**f**) Reconstructions obtained using a background model with homogeneous layers.

In the previous cases, the true layer profiles were assumed to be perfectly homogeneous and exactly known. As a final example, we consider a scenario in which the background medium exhibits inhomogeneous variations around reference values. To investigate the robustness of the proposed method under such conditions, the true background profile is constructed by adding spatially varying perturbation (noise) terms of the form ηεmReei2πrd and ησmReei2πrd to the permittivity and conductivity values of the first and second layers (m=1,2) of the configuration in the previous example, with η=0.10, thereby introducing inhomogeneous fluctuations around the reference values. The arc-shaped object with a relative permittivity of 8 and a conductivity of 0.8 S/m is embedded in this true background as seen in Figure 10a,b. For the imaging process, the background model is assumed to be composed of homogeneous layers, defined by the reference values (see Figure 10c,d). The normalized $L^{2}$-norm mismatch errors between the true and assumed background profiles are 0.07 for both relative permittivity and conductivity. The inversion results obtained with the positivity constraint are shown in Figure 10e,f. Despite some distortion in the reconstructed shape, the location and electrical parameters of the object are recovered with satisfactory accuracy. The corresponding reconstruction errors are ${NRE}_{\varepsilon }=0.08$ and ${NRE}_{\sigma }=0.40$. These results suggest that the proposed method can tolerate a moderate mismatch between the true and assumed background profiles, although such mismatch may introduce visible distortions in the reconstructed shape.

## 5. Conclusions

In this work, an efficient imaging method has been developed for objects embedded in multilayer cylindrical structures with arbitrarily shaped layer boundaries by integrating the CSI method with a recursive EE-NCS. A key aspect of the proposed approach is the incorporation of the known multilayer structure into the background model, instead of attempting to reconstruct the entire configuration simultaneously. This strategy improves reconstruction accuracy by effectively exploiting available a priori information and enables the restriction of the reconstruction domain for more focused imaging. It should be noted, however, that this treatment introduces an additional computational step compared with a straightforward free-space CSI implementation. In the proposed formulation, the background field and the inhomogeneous-background Green’s function must be computed, whereas free-space CSI only requires the analytically available free-space Green’s function. Nevertheless, the numerical results indicate that this additional computational effort is accompanied by a clear improvement in reconstruction accuracy. To mitigate the additional cost, the recursive EE-NCS formulation is employed to compute the background field and the associated Green’s function efficiently. Therefore, although the proposed method is computationally more demanding than free-space CSI, it remains substantially more efficient than inhomogeneous-background CSI implementations based on fully numerical background solvers such as MoM or FEM. Comparisons with MoM results confirm that the proposed approach provides accurate forward modeling and reliable inversion performance. Numerical results demonstrate that the method successfully reconstructs the location, shape, and electrical properties of embedded objects under various configurations, including scenarios involving a PEC core. It is also observed that the computational advantage of the proposed approach becomes more pronounced as the electrical size increases, although a gradual degradation in accuracy may be observed for electrically larger configurations. Future work will focus on evaluating the performance of the proposed method under experimental measurement conditions and extending the formulation to three-dimensional configurations.

Several limitations of the present study should be acknowledged, together with their effect on the reconstruction quality. First, the results reported here are obtained entirely from synthetic data generated by a full-wave MoM forward solver; the proposed method has not yet been validated against experimental measurements. Real measurement data would introduce model mismatch, noise, calibration errors, and uncertainties in the assumed host geometry that are not fully captured by the present synthetic study, and an experimental validation is therefore left for future work. Second, a central assumption of the proposed formulation is that the multilayer background, i.e., the number, shape, and electrical parameters of the host layers, is known a priori and can be incorporated into the background model with sufficient accuracy. This assumption is reasonable for several controlled sensing scenarios, such as cylindrical industrial components, pipes, and engineered layered structures, where the host geometry and material properties are manufactured, designed, or characterized in advance. Nevertheless, when the background is only partially known or is itself uncertain, errors in the assumed host parameters act as an additional, unmodeled contrast. This may bias or degrade the reconstruction of the embedded objects. The final numerical example indicates that the proposed method can still provide reasonable reconstructions under a moderate mismatch between the true and assumed background profiles. However, larger uncertainties in the background model are expected to have a stronger adverse effect, and jointly estimating or updating the background remains an important extension of this work. Third, the EE-NCS truncation number N affects the convergence of the background-field and background Green’s-function computations. Although larger or more complex multilayer structures generally require larger values of N, excessively large N may reduce numerical robustness because of the difficulty of evaluating higher-order cylindrical functions under standard double-precision arithmetic. Hence, the range of stable and accurate N values may become limited for highly irregular or electrically large configurations. This issue has been discussed in more detail in our previous EE-NCS studies [23,24,25,26,27], and is therefore not examined separately in the present work.

## Data Availability

The original contributions presented in this study are included in the article/Supplementary Materials. Further inquiries can be directed to the corresponding author.

## Supplementary Materials

The following supporting information can be downloaded at: https://www.mdpi.com/article/10.3390/s26134134/s1, example1.txt; example2.txt; example3.txt; example4.txt; example5.txt.

## References

[1] Kwon S., Lee S. (2016). Recent Advances in Microwave Imaging for Breast Cancer Detection. Int. J. Biomed. Imaging.

[2] Pokorny T., Redr J., Laierova H., Smahelova B., Kollar J. (2025). An Experimental 10-Port Microwave System for Brain Stroke Diagnosis—Potentials and Limitations. Sensors.

[3] Gao S., Hu D. (2026). Ground Penetrating Radar for Subsurface Utility Detection: Methods, Challenges, and Future Directions. Sensors.

[4] Amin M.G. (2013). Through-the-Wall Radar Imaging.

[5] Pastorino M., Raffetto M., Randazzo A. Reconstruction of dielectric and velocity profiles in pipelines through an electromagnetic inverse scattering technique. Proceedings of the IEEE International Conference on Imaging Systems and Techniques.

[6] Sobkiewicz P., Bieńkowski P., Błażejewski W. (2021). Microwave Non-Destructive Testing for Delamination Detection in Layered Composite Pipelines. Sensors.

[7] Fhager A., Persson M. (2007). Using a priori Data to Improve the Reconstruction of Small Objects in Microwave Tomography. IEEE Trans. Microw. Theory Tech..

[8] Gilmore C., Zakaria A., Pistorius S., LoVetri J. (2013). Microwave Imaging of Human Forearms: Pilot Study and Image Enhancement. Int. J. Biomed. Imaging.

[9] Leone G., Soldovieri F., Persico R. (2004). Microwave Imaging of Buried Object under the Distorted Born Approximation. Proceedings of the 2004 IEEE International Workshop on Imaging Systems and Techniques (IST), Stresa, Italy, 13–14 May 2004.

[10] Schenone V., Estatico C., Gragnani G.L., Pastorino M., Randazzo A., Fedeli A. (2023). Microwave-Based Subsurface Characterization through a Combined Finite Element and Variable Exponent Spaces Technique. Sensors.

[11] Yamauchi Y., Kidera S. (2024). Complex Permittivity Imaging by Incorporating Synthetic Aperture Radar and Inverse Scattering Method for Stratified Ground Medium. IEEE J. Sel. Top. Appl. Earth Obs. Remote Sens..

[12] Yamauchi Y., Kidera S. (2023). Contrast Source Inversion-Enhanced Synthetic Aperture Approach for Microwave Multilayered Subsurface Imaging. IEEE Trans. Antennas Propag..

[13] Wu Q., Huang R., Han F. (2024). 2-D EM Scattering and Inverse Scattering from Inhomogeneous Objects Straddling Multiple Subsurface Planar Layers with a Rough Surface. IEEE Geosci. Remote Sens. Lett..

[14] Gürbüz T.U., Aslanyürek B. (2015). A Two-Stage Procedure for Microwave Imaging of a Buried Dielectric along with the Randomly Rough Surface above It. Int. J. Antennas Propag..

[15] Yelkenci T. (2019). Inverse scattering related to cylindrical bodies buried in a lossy circular cylinder with resistive boundary. Frequenz.

[16] Akbari Sekehravani E., Leone G. (2023). Evaluation of the Resolution in Inverse Scattering of Dielectric Cylinders for Medical Applications. Sensors.

[17] Chen X. (2010). Subspace-based optimization method for inverse scattering problems with an inhomogeneous background medium. Inverse Probl..

[18] Konakyeri Arıcı E., Yapar A. (2019). An inverse scattering approach based on inhomogeneous medium Green’s functions for microwave imaging of brain strokes. Adv. Electromagn..

[19] Xu K., Zhong Y., Chen X., Lesselier D. (2018). A fast integral equation-based method for solving electromagnetic inverse scattering problems with inhomogeneous background. IEEE Trans. Antennas Propag..

[20] Van den Berg P.M., Kleinman R.E. (1997). A contrast source inversion method. Inverse Probl..

[21] Hirose U., Zhu P., Kidera S. (2022). Deep Learning Enhanced Contrast Source Inversion for Microwave Breast Cancer Imaging Modality. IEEE J. Electromagn. RF Microw. Med. Biol..

[22] Li Y., Zhao H., Sun H. (2023). ICSI: An Improved Contrast Source Inversion Method for Electromagnetic Inverse Scattering Problems. Opt. Express.

[23] Gürbüz T.U. (2018). Computation of Two-Dimensional Green’s Function for Arbitrary Shaped Multilayer Cylinders. IEEE Antennas Wirel. Propag. Lett..

[24] Gürbüz T.U., Aslanyürek B. (2020). An efficient recursive approach for electromagnetic scattering by arbitrary-shaped multilayer cylinders. IEEE Antennas Wirel. Propag. Lett..

[25] Gürbüz T.U. (2020). Keyfi Şekilli Silindirlere Gömülü Dielektrik Cisimlerin Etkin Bir Mikrodalga Ters Saçılma Yaklaşımı ile Görüntülenmesi. DÜMF Mühendislik Derg..

[26] Aslanyürek B., Gürbüz T.U. (2017). A Continuity-Based Series Solution for Electromagnetic Scattering by Arbitrary Shaped Multilayer Cylinders: TM Case. IEEE Trans. Antennas Propag..

[27] Aslanyürek B. (2020). TM scattering by arbitrary-shaped multilayer cylinders having embedded inhomogeneities. IEEE Antennas Wirel. Propag. Lett..

